# Fatty Acids Are Responsible for the Discrepancy of Key Aroma Compounds in Naturally Dried Red Goji Berries and Hot-Air-Dried Red Goji Berries

**DOI:** 10.3390/foods14132388

**Published:** 2025-07-06

**Authors:** Yan Zheng, Claudia Oellig, Walter Vetter, Vanessa Bauer, Yuan Liu, Yanping Chen, Yanyan Zhang

**Affiliations:** 1 Department of Food Chemistry and Analytical Chemistry, University of Hohenheim, Garbenstraße 28, D-70599 Stuttgart, Germany; yan.zheng@uni-hohenheim.de (Y.Z.);; 2 Institute of Food Chemistry, University of Hohenheim, Garbenstraße 28, D-70599 Stuttgart, Germany; 3 School of Food Science and Engineering, Ningxia University, Yinchuan 750021, China; 4 Department of Food Science & Technology, School of Agriculture & Biology, Shanghai Jiao Tong University, Dongchuan Road 800, Shanghai 200240, China; 5 Department of Flavor Chemistry, University of Hohenheim, Fruwirthstraße 12, D-70599 Stuttgart, Germany

**Keywords:** *Lycium*, goji berry, aroma, fatty acid, natural drying, hot-air drying

## Abstract

Red goji berries, reputed worldwide as “superfruit”, are commonly marketed after natural drying or hot-air drying. A sensomics approach was applied to the aroma analysis of red goji berries under two drying methods. Fifty-two aroma-active compounds were screened and identified by aroma extract dilution analysis (AEDA) coupled with gas chromatography with olfactometry (GC/O). The contents and the odor activity values (OAVs) of 49 aroma-active compounds were determined. Acetic acid was the predominant aroma compounds in both berries. Meanwhile, the key aroma compounds in both berries were (*E*)-2-nonenal, (*Z*)-4-heptenal, 3-methyl-2,4-nonanedione, hexanal, etc., which were lipid derivatives. Natural drying promoted the formation of some aldehydes that exhibited green and fatty notes. Hot-air drying facilitated the production of ketones with hay-like and cooked apple-like odor attributes due to the thermal reaction. The fatty acid patterns between naturally dried and hot-air-dried red goji berries differed not significantly and were dominated by linoleic acid, oleic acid, palmitic acid, etc. The knowledge of the impacts of different drying processes on the aroma quality in red goji berries is beneficial for the quality control and optimization of dried red goji berries.

## 1. Introduction

Red goji berries are the fruits of some species of *Lycium* plants, i.e., perennial, deciduous shrubs that belong to the family of *Solanaceae* plants [1]. Nowadays, they are mainly cultivated in China, Mongolia, and the Mediterranean region (Europe and Africa) [2,3]. Goji berries are appraised as medicinal and functional foods, and their consumption may moderate Parkinson’s disease, dizziness, fatigue, and sleep problems [4,5]. These benefits of goji berry consumption were attributed to their richness in valuable nutrients, including polyphenols, polysaccharides, carotenoids, sterols, and fatty acids [1,6,7]. Due to their nutritional benefits and medicinal values, fruits (eaten raw or dried), seeds, root barks, and processed products, such as dietary supplements, beverages, medicines, and food incorporations, are marketed worldwide [4].

However, fresh red goji berries with tender tissues and high moisture contents (approximately 75–85%) are sensitive to microbial spoilage and deterioration [8]. Drying is an essential process for extending the storage of red goji berries. The commonly applied methods for red goji berries are natural drying and hot-air drying [9,10]. For natural drying, the fresh goji berries are laid thinly and flatly under the sunshine after harvesting [10]. Considering weather uncertainties, possible contaminations, the long drying time, and the limitation of the drying scale during natural drying, hot-air drying has become an alternative method, as it leads to a rapid decrement in the surface moisture without a loss in the quality attributes of goji berries [10]. Regardless of the drying process, the moisture content in commercial-dried goji berries is reduced to less than 13% in China [11]. In the U.S. and the EU, no specific regulation mentions the moisture content in commercial-dried goji berries. However, the general limitation of moisture content in dried fruits is below 25% [12].

Aroma is the foremost aspect that reflects the properties of goji products and drives consumption decisions [13]. Therefore, determining aroma-active compounds is of great importance for commercial goji berry samples and products. Accordingly, the volatiles, including aroma-active compounds, were determined in the fresh red goji berries [14,15], pulsed vacuum-dried red goji berries [8], fermented goji juices [16], and goji wines [17]. A recent investigation reported that sixty-three volatiles were identified in dried goji berries (water contents <10%) [18], which included sixteen alcohols, thirteen aldehydes, ten esters, seven ketones, five terpenes, five carboxylic acids, two ethers, two nitriles, two pyrazines, and one alkene. However, not all volatiles are aroma-active compounds. The subsequent report detected 45 and 39 aroma-active compounds in commercial-dried red and black goji berries, including (*E*)-*β*-damascenone, (*E*)-2-nonenal, 1-octen-3-one, (*E*,*E*)-2,4-nonadienal, (*E*)-*β*-ionone, etc. [19] However, no systematic aroma analysis has been applied to the comparison of naturally dried and hot-air-dried red goji berries. 

The volatiles in dried goji berries are dominated by alcohols, aldehydes, and ketones [18,19,20]. These compounds are the degradation products of unsaturated fatty acids, with linoleic acid (18:2*n-6*), oleic acid (18:1*n-9*), and *γ*-linolenic acid (18:3*n-6*) being the most prominent ones in red goji berries [7]. Yet, the impact of both drying processes on the fatty acids of red goji berries is still unclear. Furan fatty acids (FuFAs) belong to the group of fatty acids and are omnipresent in various plants [21]. Their detection requires more sophisticated methods than those applied for analyzing conventional fatty acids, since these sensitive antioxidants are easily degraded [21]. FuFAs in goji berries were recently analyzed due to their key role as the precursor of the key aroma compound, i.e., 3-methyl-2,4-nonadienone, in goji berries [15]. However, the effect of natural drying and hot-air drying on FuFAs has never been reported.

A sensomics approach is an advanced method of aroma analysis. It is chosen to identify the key aroma-active compounds of a given food within the bulk of overall volatiles [13]. This method combined sensory with instrumental analysis, e.g., gas chromatography with olfactory (GC/O), gas chromatography with mass spectrometry (GC/MS), and multidimensional gas chromatography, to decode the key aroma compounds. The results are further verified by using aroma recombination [13]. This approach has already been successfully applied to numerous foods, such as goji wines [17], licorice [22], and *Toona sinensis* [23], etc. In detail, this application involved (i) the isolation of volatile compounds by solvent-assisted flavor evaporation (SAFE); (ii) odorant screening by aroma extract dilution analysis (AEDA) via GC/O; (iii) the identification of aroma-relevant compounds based on retention indices (RIs) on both DB-FFAP and DB-5 capillary columns, odor quality, and mass spectra; (iv) the quantitation and calculation of odor activity values (OAVs) of these aroma-active compounds; (v) a sensory evaluation of aroma recombination. However, there is still a lack of relative applications on naturally dried red goji berries and hot-air-dried red goji berries. Therefore, the aim of this study is to investigate the influence of drying methods on the aroma profiles and provide strong evidence of quality control and food supervision of goji products. Due to the relevance of fatty acids as the potential precursors of important aroma compounds, the fatty acid pattern could also be analyzed for possible changes in both drying methods.

## 2. Materials and Methods

### 2.1. Materials

The naturally dried red goji berries (NG, 10 kg) and hot-air-dried red goji berries (HG, 10 kg) were the same variety, collected in the same cultivation farm and at same harvest time, and then commercially produced by Ningxia Zhongning County Qixiangyun Wolfberry Technology (Ningxia, China). All commercial-dried goji berries complied with market regulations, maintaining final moisture contents below 13% [11]. The berries were frozen in liquid nitrogen and powdered in a Spex CertiPrep 6800 Freezer/Mill (Spex, Metuchen, NJ, USA) with twice the amount of anhydrous sodium sulfate to remove the water in the goji berries and make fine powder. The goji powder was well-packed in plastic bags and stored at −20 °C before use.

### 2.2. Chemicals and References

Methyl hexanoate (99%), phenol (≥99%), phenylacetaldehyde (98%), 3-phenylpropanoic acid (99%), and vanillin (99%) were obtained from Acros Organics (Thermo Fisher Scientific, Nidderau, Germany); butanoic acid (99%), (*E*,*E*)-2,4-decadienal (90% mixture isomers), 2-ethyl-3,5-dimethylpyrazine (99% mixture isomers), 4-ethylphenol (97%), heptanoic acid (98%), hexanal (98%), (*E*)-3-hexenoic acid (97%), 2-methoxyphenol (98%), 2-methylbutanoic acid (98%), 3-methyl-1-butanol (99%), phenylacetic acid (99%), pentyl hexanoate (≥98%), and *α*-terpineol (96%) were purchased from Alfa Aesar (Karlsruhe, Germany); 3-isobutyl-2-methoxypyrazine (99.6%), (*E*,*Z*)-2,6-nonadienal (96% mixture isomers), and 1-octen-3-one (97.7%) were ordered from BLDpharm (Karlsruhe, Germany); dichloromethane (for HPLC) was commercially obtained from Carl Roth (Karlsruhe, Germany); acetic acid (≥99%) and nonanoic acid (>97%) were delivered by Fluka (Neu-Ulm, Germany); dimethyl trisulfide (98%), (*Z*)-4-heptenal (95%), 3-hydroxy-4,5-dimethyl-2(5*H*)-furanone (97.5%), 3-methylbutanoic acid (99%), 2-methoxy-4-vinylphenol (98%), (*E*,*E*)-2,4-nonadienal (85%), 1-octen-3-ol (98%), and 2-undecanone (98%) were provided by J&K Scientific (Shanghai, China); decanal (97%) and eugenol (Pharmacopoeia Europaea, Ph. Eur.) were from Merck (Darmstadt, Germany); (*E*)-*β*-damascenone (1.1~1.4%), (*E*)-2-decenal (>95%), 1,2-dichlorobenzene (99%), ethyl butanoate (99%), 4-ethyl-2-methoxyphenol (98%), (*E*,*E*)-2,4-heptadienal (90%), 4-hydroxy-2,5-dimethyl-3(2*H*)-furanone (99%), and octanal (99%) were purchased from Sigma-Aldrich Chemie (Taufkirchen, Germany); 2-ethylphenol (>98%), (*E*)-2-heptenal (>95%), 3-mercapto-3-methyl-1-butanol (>98%), 3-methyl-2,4-nonanedione (>97%), nonanal (95%), (*E*)-2-nonenal (>95%), (*E*)-2-octenal (>96%), and (*E*)-2-undecenal (>93%) were delivered by TCI (Eschborn, Germany); ethanol, *n*-hexane, and methanol (all HPLC degree) were obtained from Th. Geyer (Renningen, Germany). Liquid nitrogen, hydrogen (99.999%), and nitrogen (99.95%) were ordered from Westfalen (Stuttgart, Germany). Internal standards for fatty acid analysis, myristic acid ethyl ester (14:0-EE) and 10,11-dichloroundecanoic acid (DC-11:0), were synthesized according to Thurnhofer et al. [24].

The stable isotopically labeled references, *d_2_*-butanoic acid, *d_2_*-3-methylbutanoic acid, *d_16_*-octanal, *d_2_*-phenylacetic acid, and *d_3_*-vanillin, were obtained from Prof. Dr. Michael Granvogl (Department of Food Chemistry and Analysis Chemistry, University of Hohenheim).

### 2.3. Analysis of Aroma Compounds

#### 2.3.1. Isolation of the Volatiles

A portion of goji berry powder (30 g, containing 10 g of goji berries and 20 g of sodium sulfate) was magnetically extracted with dichloromethane (120 mL) overnight at room temperature. The extract was filtered. The residue was washed again for 1 h with dichloromethane (2 × 30 mL) and filtered. The organic solution extracts were combined, and the volatiles were isolated from non-volatiles at 40 °C using the SAFE apparatus [25]. The distillate was dried over anhydrous sodium sulfate and concentrated to ~200 µL using a Vigreux column (50 cm × 1 cm) and a microdistillation apparatus. All samples were prepared and evaluated in triplicate.

#### 2.3.2. Gas Chromatography with Olfactory (GC/O)

GC/O analysis was carried out using a type 5160 gas chromatograph (Carlo Erba Instruments, Milano, Italy), as previously described [17]. Two different polarities of fused-silica capillary columns (DB-5 and DB-FFAP, both 30 m × 0.32 mm i.d., 0.25 µm film thickness) (J&W Scientific; Agilent Technologies, Waldbronn, Germany) were used. The beginning of the analytical column was equipped with a deactivated fused silica pre-column (5 m × 0.32 mm i.d., 0.25 µm film thickness; J&W Scientific; Agilent Technologies, Waldbronn, Germany), and the end of the analytical column was connected to a Y-shaped splitter (Agilent Technologies, Santa Clara, CA, USA) to divide the effluent into two equal parts. These two equal parts were performed with two equal-length deactivated fused silica capillary columns (50 cm × 0.32 mm i.d.), which equally distributed the effluent between the flame ionization detector (FID, 230 °C) and the sniffing port (230 °C), respectively. After the injection of the sample (2 µL), the temperature was held at 40 °C for 2 min. Then, the oven temperature was ramped at 6 °C/min to 230 °C and held at 230 °C for 5 min. A trained person placed their nose above the sniffing port to evaluate the effluent and marked the odorous regions and odor quality on the FID chromatography in the recorder. Each RI of the odorous region was calculated from the retention time and the adjacent retention times of the series of *n*-alkanes (C_6_-C_19_ for DB-5 and C_6_-C_26_ for DB-FFAP). The samples were evaluated by panelists (n = 3).

#### 2.3.3. Aroma Extract Dilution Analysis (AEDA)

The distillate (~200 µL) was first adjusted to 1 mL and then stepwise diluted 1:1 (*v/v*) with dichloromethane [26]. Each dilution was analyzed by GC/O on the DB-FFAP column. Each odorous region was assigned a flavor dilution (FD) factor, which was assigned to be the highest dilution. To compare the goji berry samples with two different drying methods, the sample weights (30 g of powder), distillate concentrations (1 mL), and injection volumes (2 µL) were measured under the same conditions.

#### 2.3.4. Gas Chromatography with Mass Spectrometry (GC/MS) for Aroma Analysis

Thermo Scientific trace 1300 gas chromatography combined with a Thermo ISO 7000 single quadrupole mass spectrometer (Dreieich, Germany) was used according to Zheng et al. [17]. The sample (1 µL) was injected into the capillary columns, DB-FFAP column, or DB-5 column (both 30 m × 0.25 mm i.d., 0.25 μm film thickness; both J&W Scientific; Agilent Technologies, Waldbronn, Germany) via a Thermo TriPlus RSH autosampler (Dreieich, Germany). The carrier gas helium was transported at 1.6 mL/min. The oven temperature was programmed as mentioned in Section 2.3.2. The sample was analyzed in electron ionization mode (GC/EI-MS) at 70 eV, and mass spectra were recorded in full scan mode (*m/z* 35–300).

#### 2.3.5. Identification of Aroma-Active Compounds

Odor-active compounds were assigned through RIs, odor quality, and odor intensity at the sniffing port compared to the height of the FID signal on two capillary columns (DB-5 and DB-FFAP capillary columns), and GC/EI-MS spectra were used in combination with data in the Leibniz-LSB@TUM Odorant Database, the previous literature, and the authentic references.

#### 2.3.6. Determination of the Contents of Aroma-Active Compounds

The precise quantitation was carried out by using stable isotope dilution assays (SIDAs). The rest of the aroma compounds were semi-quantified by using the internal standard method if the relevant isotope-labeled references were unavailable. In general, the internal standard (1,2-dichlorobenzene, *d_2_*-butanoic acid, *d_2_*-3-methylbutanoic acid, *d_16_*-octanal, *d_2_*-phenylacetic acid, and *d_3_*-vanillin) and the analytes were dissolved in dichloromethane for the establishment of the calibration curves. Calibration factors were determined by analyzing the concentration ratios (1:5, 1:3, 1:1, 3:1, and 5:1; *x*-axis) of internal standards and analytes and the responding peak area ratios (*y*-axis) under selected ions (Table 1). Considering the data estimated from preliminary experiments, the reference (2–50 µg) in dichloromethane was equilibrated for 30 min with different amounts of goji berry powder (10–150 g). The isolation of volatiles was performed as mentioned in Section 2.3.1.

#### 2.3.7. Sensory Assessment

After receiving the approval of the Ethics Committee of the University of Hohenheim (approval time was April 2024), sensory assessment was carried out for the original samples and the aroma recombinations by 10 weekly trained panelists (including 2 males and 8 females, 23–32 years old) at room temperature. The participants voluntarily participated prior to the sensory assessment. Moreover, all the concentrations of the used aroma-active compounds were below the concentrations that were approved by the European Union and European Food Safety Authority, which referred to the websites: https://eur-lex.europa.eu/legal-content/EN/TXT/?uri=CELEX:32012R0872v (accessed on 16 April 2024); https://pubchem.ncbi.nlm.nih.gov/ (accessed on 16 April 2024).

##### Aroma Profile Analysis (APA)

Original NG and HG (10 g each) were placed into two closed 28 mL brown glass bottles at room temperature, respectively. The reference odorants were selected as 3-methyl-2,4-nonanedione (hay-like), (*E*)-2-nonenal (green and fatty), hexanal (green), 1-octen-3-one (mushroom-like), (*E*)-*β*-damascenone (cooked apple-like), acetic acid (vinegar-like), ethyl butanoate (fruity), and nonanal (citrus-like). Each reference odorant was dissolved in ethanol and diluted in water at a concentration of 100-fold of the odor threshold (OT) in the water matrix. The final concentration of ethanol was below 990,000 µg/kg in the water matrix. A seven-point scale (from 0 to 3, 0.5 increments) was used for rating the intensity of these descriptors (from no intensity to strong intensity). Each result of the odor description for the APA was calculated as the average of the scores obtained from the 10 panelists.

##### Aroma Recombination

To get the odorless goji matrix, ~50 g of the original whole dried red goji berries were magnetically extracted with dichloromethane (500 mL) at room temperature for at least one week. After removing the solvent, the residue was washed thrice with dichloromethane (150 mL) for one day. The finely washed residue was set thinly and plainly in the hood for at least two months to remove the odor. The OAVs of aroma-active compounds in dried red goji berries were calculated by dividing determined concentrations by their respective odor thresholds (OTs) in water, which have limited effects for dried samples. All aroma-active compounds with OAVs > 1 were diluted in water (10 mL in total) at the determined contents in the original samples. A model of recombination containing the odorless matrix (10 g) and the aroma-active compounds was finely mixed and kept in equilibrium for one day in the sealed 28 mL brown glass bottle. The orthonasal evaluation of the recombination test was performed as mentioned in the Aroma Profile Analysis (APA) Section.

### 2.4. Analysis of Fatty Acids

#### 2.4.1. Transesterification of Fatty Acids

The commercial NG and HG (not freezer-milled) were ground at room temperature by a laboratory mill (IKA A11; Staufen, Germany). About 0.5 g of the powder was accurately weighed in an 8 mL glass test tube with a screw cap, and then the cold extraction with *n*-hexane (6 mL) in the ultrasonic bath was performed three times, as mentioned in a previous study [7]. The organic solvents were collected, combined, and then concentrated under reduced pressure (35 °C, 298 mbar). The final extract was adjusted to 1 mL prior to use. An aliquot of the extract (300 µL) and the first internal standard solution (DC-11:0, 3 µL, 1 µg/mL in *n*-hexane) were placed in an 8 mL test tube, dried under gentle nitrogen steam, and then supplemented with sulfuric methanol solution (1%, *v/v*, 1 mL). The tube was placed in the sand bath (80 °C, 1 h) for transesterification. After cooling in the ice bath, the tube was filled with distilled water (1 mL), saturated sodium chloride solution (1 mL), and *n*-hexane (2 mL). The organic phase (1.5 mL) was collected and supplemented with the second internal standard (14:0-EE, 3 µL, 1 µg/mL in *n*-hexane). All samples were prepared in duplicate.

#### 2.4.2. Gas Chromatography with Mass Spectrometry (GC/MS) for Fatty Acid Analysis

After their conversion into the corresponding methyl esters, fatty acids were analyzed on 5890 series II Plus and 5972 GC/MS combo 140 (Hewlett-Packard/Agilent, Waldbronn, Germany) equipped with an Rtx-2330 capillary column (60 m × 0.25 mm i.d., 0.1 µm film thickness; Restek, Bellefonte, PA, USA) [7]. Samples (1 µL) were injected by a 7673 autosampler (Hewlett-Packard/Agilent, Waldbronn, Germany). The GC oven temperature was kept at 60 °C for 1 min, followed by heating rates to 150 °C at 6 °C/min, 190 °C at 4 °C/min, and 250 °C at 7 °C/min, which was held for 7 min [7]. A constant flow (helium) was set at 1 mL/min. GC/MS data were generated in full scan mode (*m/z* 50–500) and selected ion monitoring (SIM) mode as described before [24].

### 2.5. Statistical Analysis

The significant differences between the two samples were analyzed by an independent two-sample *t*-test and paired *t*-test by using Excel 2016 (function TDIST; Microsoft, Redmond, WA, USA).

## 3. Results and Discussion

### 3.1. Sensory Analysis of Natural-Dried and Hot-Air-Dried Red Goji Berries

**Figure 1:**
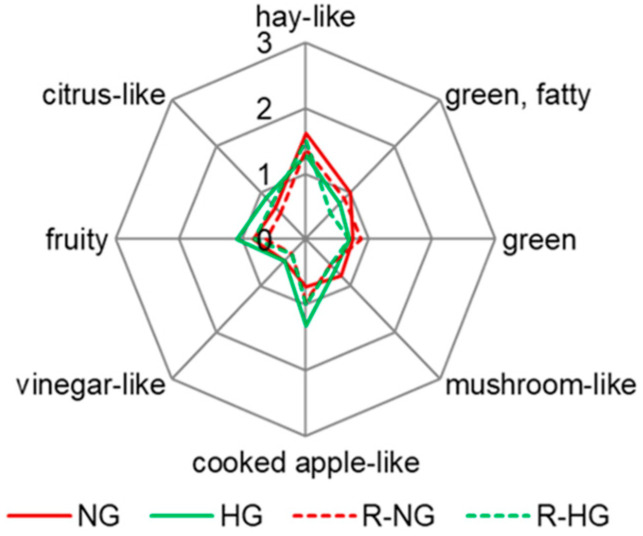
The radar plot of the sensory analysis of dried red goji berries and the recombinants. NG, naturally dried red goji berries (red line); HG, hot-air-dried red goji berries (green line); R-NG, the aroma recombinant of naturally dried red goji berries (red dash line); R-HG, the aroma recombinant of hot-air-dried red goji berries (green dash line).

The overall aroma profiles of the naturally dried goji berries (NG) and hot-air-dried goji berries (HG) were similar (Figure 1, red and green lines, no significant difference with *p* > 0.05). Specifically, both were characterized by a high-intensity hay-like odor impression and a low-intensity vinegar-like odor impression (Figure 1, red and green lines). Also, the flavor notes were balanced without a clear dominance of one flavor sensation. The minor differences were that NG exhibited more intensive hay-like, green and fatty, green, and mushroom-like odor notes, while HG was richer in cooked apple-like, fruity, and citrus-like odor notes (Figure 1, red and green lines).

### 3.2. Identification of Aroma-Active Compounds in Both Dried Red Goji Berries

**Figure 2:**
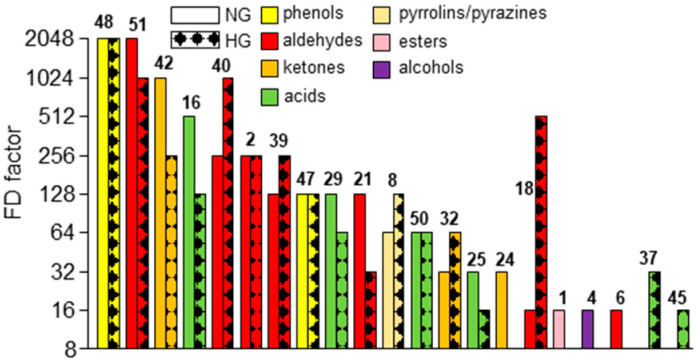
Flavor dilution (FD) factors (compounds with FD factors ≥16 are displayed) of naturally dried and hot-air-dried red goji berries. The numbering refers to Table 1.

Orthonasally assessed on a strip of filter paper, the concentrated SAFE distillates of both dried red goji berry samples (Section 2.3.1) showed the characteristic odor of the original sample (before extraction). The application of GC/FID, GC/O (Section 2.3.2), and AEDA (Section 2.3.3) enabled the assignment of 50 detected odor-active regions in NG and 47 in HG (in total, 52, Table 1). Both samples had 45 compounds in common, while 5 additional ones were unique for NG (**1**, **24**, **27**, **34**, **41**) and 2 for HG (**19** and **44**). Slight but remarkable differences were also observed for the compounds with the most intense smells (Figure 2). Specifically, 2-methoxy-4-vinylphenol (**48**) and vanillin (**51**) (both FD = 2048) dominated in NG, where they contributed to the smoky, clove-like, and vanilla-like, sweet smells (Table 1). Compared to that, HG was dominated by 2-methoxy-4-vinylphenol (**48**, FD = 2048), followed by *trans*-4,5-epoxy-(*E*)-2-decenal (**40**, FD = 1024) and vanillin (**51**, FD = 1024). Accordingly, vanillin (**51**) was more and *trans*-4,5-epoxy-(*E*)-2-decenal (**40**) was less relevant in NG (Figure 2). Most importantly, these and other phenols with low FD factors (**36**, **41**, **43**, **44**, **46**, **47**, and **48**) were absent in fresh red goji berries [8,14,15], and associated with a phenolic and smoky aroma. Therefore, these phenols marked the characteristic aroma differences between dried and fresh red goji berries. This may indicate that they were released from glycosylated precursors during drying.

The major structural group of the identified aroma-active compounds was aldehydes (n = 16) formed via the degradation of unsaturated fatty acids. A high variety was also observed for acids (n = 8), phenols (n = 7), ketones (n = 6), alcohols (n = 4), and esters (n = 4) (Table 1).

In agreement with the wide variety, the transformation products of unsaturated fatty acids like hexanal (**2**), *trans*- and *cis*-4,5-epoxy-(*E*)-2-decenal (**40**, **39**), and decanal (**18**) [27,28] had an essential influence on the flavor of dried red goji berries (Section 3.3).

Compared to that, the six ketones played a crucial role in the aroma profiles in NG and HG, and three of them were assigned as key aroma compounds in NG and HG (see Section 3.3. These six ketones originated from four different precursor families. First, the relevant aroma-active compounds, 4-hydroxy-2,5-dimethyl-3(2*H*)-furanone (furaneol, **42**) and 3-hydroxy-4,5-dimethyl-2(5*H*)-furanone (sotolon, **49**), contributed to the caramel-like, smoky, and fenugreek-like odor notes [29,30]. The former one (**42**) naturally occurs in fruits and vegetables but is also thermally produced in processed food [29,30]. The known precursors are hexoses and pentoses that contribute to the heat of the Maillard reaction [29]. By contrast, 3-hydroxy-4,5-dimethyl-2(5*H*)-furanone (49) was found to be thermally induced from 4-hydroxyisoleucine with oxidative deamination or from ethanol and ascorbic acid [31,32,33]. Due to their extremely low OTs in water, 4-hydroxy-2,5-dimethyl-3(2*H*)-furanone (**42**) and 3-hydroxy-4,5-dimethyl-2(5*H*)-furanone (**49**) are known for noticeable contributions to the overall aroma of food [29,30].

Second, (*E*)-*β*-damascenone (**35**) belongs to the family of C_13_-norisoprenoids and is known to be formed by the acid-catalyzed hydrolysis of plant-derived apocarotenoids [34]. In plants, apocarotenoids are commonly stored as the aglycon of glycosides, which can be degraded into (*E*)-*β*-damascenone both chemically at higher temperatures and low pH values and enzymatically by glycosidases [34].

Third, 3-methyl-2,4-nonanedione (**32**) is known to be formed from *β*,*β*’-dimethyl-substituted furan fatty acids (D-FuFAs) [35], and for this reason, this aspect was investigated more in Section 3.5.

Finally, fourth, 1-octen-3-one (**7**) and 2-undecanone (**24**) are most likely the degradation products of fatty acids [36].

### 3.3. (Semi-)Quantitation of Aroma-Active Compounds and Calculation of OAVs

The contributions of 47 of the 52 (5 structures could not be unequivocally verified by GC/MS) aroma-active compounds to the overall aroma profiles were determined in NG and HG (Table 2). Five (butanoic acid, 3-methylbutanoic acid, octanal, phenylacetic acid, and vanillin) of the forty-seven compounds were quantified exactly by using their respective stable isotope-labeled references with specific quantifier ions. Due to several stable isotope-labeled references being very expensive and difficult to obtain, the contents of the rest aroma compounds were semi-quantified by an internal standard (1,2-dichlorobenzene) with specific quantifier ions (Table 2). Both aroma extracts were dominated by acetic acid (**16**), followed by several aldehydes, e.g., (*E*)-2-nonenal (**21**), hexanal (**2**), octanal (**6**), and decanal (**18**). Many acids were detected in high concentrations, including nonanoic acid (**45**), 2- and 3-methylbutanoic acid (**29**), and heptanoic acid (**37**). Remarkably, alcohols, such as 3-methyl-1-butanol (**4**) and 1-octanol (**22**) (see Section 3.6), were also abundant in both dried red goji berries.

The OAV indicated the presence of 23 aroma-active compounds in NG and HG in the water matrix (OAVs ≥ 1, Table 3). Specifically, these 23 aroma-active compounds can be smelled by the human nose and thus play a key role in the overall aroma profiles of foods. Compound-specific OAVs verified that ketones and aldehydes were the key aroma-active compounds in NG and HG samples (Table 3). These aroma-active compounds contributed fatty, green, hay-like, mushroom-like, citrus-like, metallic, and cooked apple-like odor impressions to the overall aroma profile of the dried red goji berries. Several trace aroma-active compounds, e.g., (*Z*)-4-heptenal (**5**), *cis*-4,5-epoxy-(*E*)-2-decenal (**39**), 2-methoxyphenol (**36**), and 2-ethyl-3,5-dimethylpyrazine (**13**), were only present with low contents but due to their extremely low OTs, they could be perceived in both dried red goji berry samples. These four compounds had not been reported before in the comprehensive volatile analysis of goji berries [20,37], and their detection was only possible using sophisticated instrumental analysis, but they were directly perceived by GC/O in our study.

### 3.4. Aroma Recombination of Both Dried Red Goji Berries

All aroma-active compounds with OAVs ≥ 1 were combined with the contents determined in the two original samples (NG and HG) and diluted in water. For this purpose, the odorless matrix was mixed with the resulting aroma recombination solution (1:1) to simulate the atmosphere of the original samples. Compared to the original NG sample, the aroma recombinant of NG (R-NG) deviated less from the original sample, and green and cooked apple-like notes were even more intense (Figure 1, red and dashed red lines). By contrast, the aroma recombinant sample of HG (R-HG) was weaker in all aroma impressions, especially in cooked apple-like, citrus-like, as well as green and fatty odor notes (Figure 1, green and dashed green lines).

Despite these slight deviations, the overall aroma profiles of the recombinants were similar to the respective original samples (*p* > 0.05). This confirmed that the aroma-active compounds with OAVs ≥ 1 were the key aroma-active compounds in the respective samples.

### 3.5. Comparison of Fatty Acids in NG and HG

**Figure 3:**
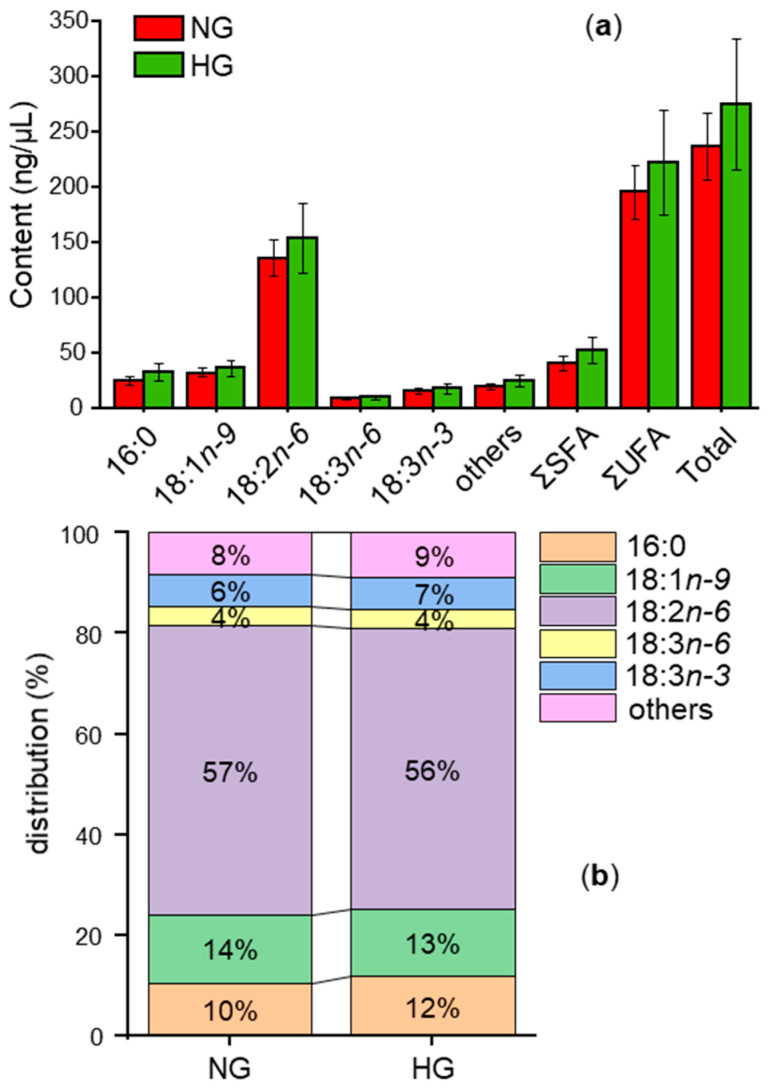
Fatty acid patterns of naturally dried red goji berries (NG) and hot-air-dried red goji berries (HG) in (**a**) content level and (**b**) percentage level.

Most of the key aroma-active compounds in dried red goji berries were generated from fatty acids. Thus, the fatty acids (on a fresh weight basis) were analyzed after their conversion into fatty acid methyl esters (FAMEs, Section 2.4.1). NG and HG both featured 21 fatty acids with linoleic acid (18:2*n-6*, ~56–58%) being predominant, followed by oleic acid (18:1*n-9c*, ~13–14%), palmitic acid (16:0, ~11–12%), *α*-linolenic acid (18:3*n-3*, ~6.3–6.5%), *γ*-linolenic acid (18:3*n-6*, ~3.5–3.9%), and palmitoleic acid (16:1*n-7*, ~0.44–0.59%) (Table 4, Figure 3). The results are consistent with the previous investigations [7].

There were no significant differences in the contents of the total fatty acids and the major fatty acids between NG and HG (Table 4, Figure 3). This agrees with the fact that only a small share of fatty acids is usually transferred into aroma compounds and that this share is difficult to determine due to the natural variation of fatty acids in samples, along with the analytical precision. Accordingly, the differences in the aroma profiles of NG and HG were most likely due to the different drying processes of NG and HG.

**Figure 4:**
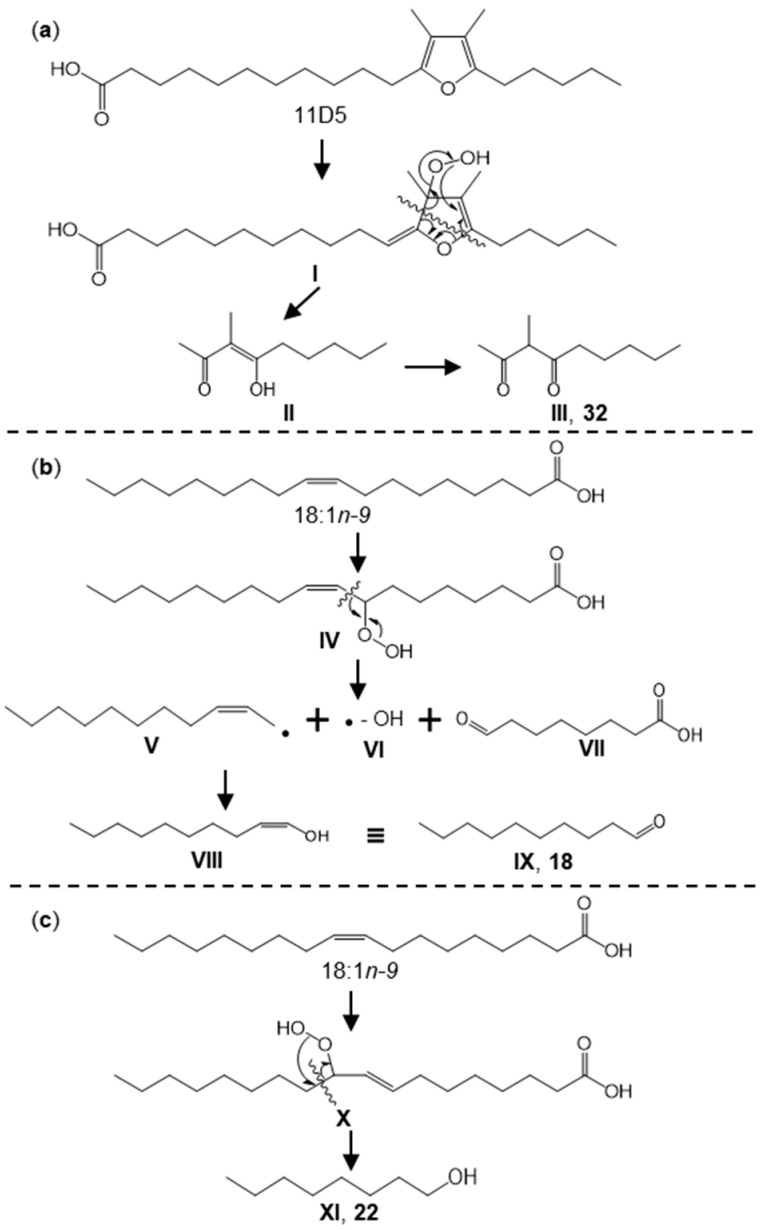
The possible biosynthesis pathways of (**a**) 3-methyl-2,4-nonanedione (**32**), (**b**) decanal (**18**), and (**c**) 1-octanol (**22**) according to ref. [36,38].

Due to the detection of 3-methyl-2,4-nonanedione (**32**), we additionally determined FuFAs in NG and HG (see Supplementary Materials). The formation of 3-methyl-2,4-nonanedione (**32**, **III** in Figure 4a) is known to originate from 11-(3,4-dimethyl-5-pentylfuran-2-yl)-undecanoic acid (11D5) after oxidation (**I** in Figure 4a). In HG, 11D5 (determined after transesterification as the methyl ester, Figure S1a) was unequivocally detected but below the limit of quantitation (LOQ), while it was not detected in NG. The higher content of 3-methyl-2,4-nonanedione (**32**) and the detection of 11D5 in HG indicated that the FuFA was less degraded by hot-air drying (HG), and this process was also less susceptible to a loss of aroma-active compounds like **32**.

The dedicated GC/MS analysis on FuFAs also enabled the detection of a small peak in NG, which was tentatively assigned to 9-(5-pentylfuran-2-yl)-nonanoic acid methyl ester (9F5-ME) based on the molecular ion, diagnostic fragment ions (Figure S1b), and retention time (20.79 min) compared to the retention time of other FuFAs mentioned in Vetter et al. [39]. Non-methyl-substituted FuFAs are not natural fatty acids but lipid oxidation products formed from polyunsaturated fatty acids [39,40]. Although 9F5 could not be quantified, its higher relevance in NG was further confirmed by the fact that lipid oxidation (and most likely photooxidation) played a more prominent role than in HG. This was further supported by the absence of 11D5 in NG.

### 3.6. Comparison of Key Aroma-Active Compounds in NG and HG

**Figure 5:**
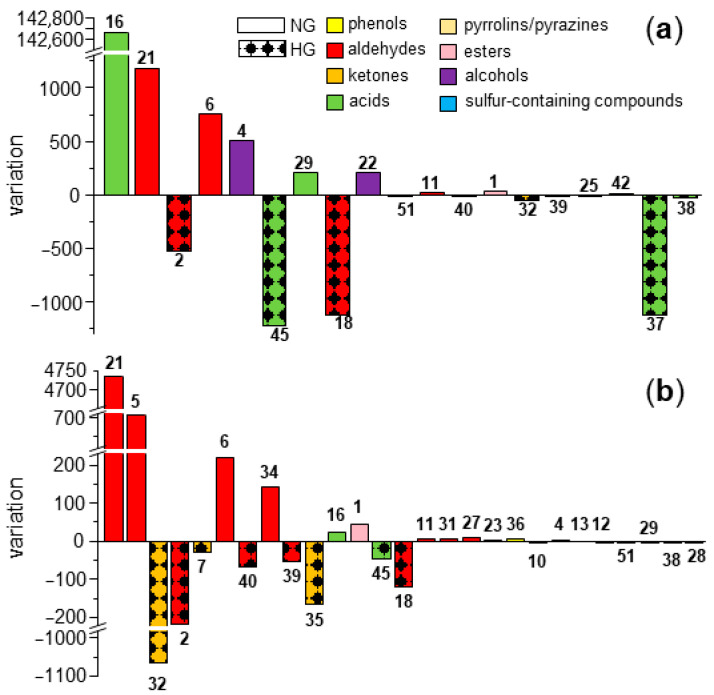
Variations in (**a**) the contents (compounds with contents ≥ 20 µg/kg are displayed) in NG minus those in HG and (**b**) OAVs (compounds with OAVs ≥ 1 are displayed) in NG minus those in HG. The numbering refers to Table 1.

While NG and HG had similar overall aroma profiles and aroma compositions (Figure 1 and Table 1), they varied in content (Table 2 and Figure 5a) and OAV levels (Table 3 and Figure 5b). First, the total content of aroma-active compounds was lower in HG than in NG, primarily because acetic acid (16) was much more abundant in NG. A considerably high proportion (%) was previously reported in dried red goji berries [41] and significantly increased after pulsed vacuum drying [8]. Excluding acetic acid, aroma-active compounds were more prominent in HG than in NG. This indicated a higher loss of (aroma-active) volatiles during the longer period in the natural drying process.

Compared to fresh red goji berries, many aldehydes and ketones were less abundant in HG and NG [15]. The OAVs of most key aroma-active aldehydes (**21**, **5**, **6**, **34**, **11**, **31**, **27**, and **23**) were ~1.2- to 7.9-fold higher in NG than in HG (Figure 5b). These aldehydes elicit fatty, green, citrus-like, cooked potato-like, and cucumber-like odor notes. They are known *β*-cleavage products of the hydroperoxides of monounsaturated (palmitoleic acid and oleic acid) and polyunsaturated fatty acids (linoleic acid and *γ*-linolenic acid) [28,36,42,43]. The higher abundance of these aldehydes in NG might be due to the enzyme-catalyzed and/or chemical-induced (autoxidation and photooxidation) oxidation of unsaturated fatty acid processes triggered by sunlight and long drying times (Table 5).

Only a few key aroma-active aldehydes (**2**, **18**, **39**, **40**) were slightly less abundant in NG than in HG (Figure 5). Hexanal (2) commonly occurs in plants and is known to be generated from linoleic acid [42]. However, unlike many other plants, red goji berries also feature *γ*-linolenic acid (Table 4). Although its levels were lower than linoleic acid, it could also contribute to the formation of hexanal due to its more pronounced sensitivity to oxidation (Table 5). Similarly, the 13-hydroperoxide of linoleic acid is a possible precursor of *trans*- and *cis*-4,5-epoxy-(*E*)-2-decenal (Table 5) [27]. The formation of decanal was rarely reported previously. Given the long, saturated alkyl chain, it is unlikely to be formed from PUFAs. The most likely formation route is via the 8-hydroperoxide of oleic acid (Figure 4b, **IV**; Table 5). Subsequent *β*-cleavage leads to 8-oxo-octanoic acid (**VII**) along with two radicals, i.e., 1-decenyl (**V**) and hydroxyl (**VI**) radicals. The combination of **VI** and **VIII** leads to 1-decen-1-ol (**VIII**), which stabilizes into the tautomeric decanal (**18**; **IX**). Such a mechanism was proposed by Frankel [36]. The same mechanism is proposed for 1-octanol (**22**; **XI**), i.e., the formation of the 10-hydroperoxide of oleic (**X**), with the formation of an octanyl radical, which reacts with the released hydroxyl radical (Figure 4c) [44]. The higher relevance of the four aldehydes (**2**, **18**, **39**, **40**) in HG might be the result of a lower level of photooxidation and the promotion of autooxidation during hot-air drying at moderate temperatures (typically 45–70 °C for HG [9,10,45,46]) (Table 5).

Ethyl butanoate (**1**), a common aroma compound in fruits that is responsible for the fruity aroma of fruits (data in Leibniz-LSB@TUM Odorant Database). It was not reported in fresh red goji berries [15], but it was the only key aroma-active ester in NG and HG. The content was richer in NG than in HG. Also, this straight-chain fatty acid ester was suggested to be generated from fatty acids through *β*-oxidation [47].

## 4. Conclusions

The ingredients and parameters of the commercial drying process are not open to the public, and the influences of drying on aroma-active compounds in red goji berries are still unclear. Our study detected 52 aroma-active compounds in both dried red goji berry samples. The (semi-)quantitation of 47 aroma-active compounds and the calculation of their OAVs enabled us to determine the key aroma-active compounds that surpassed their OTs. The assigned 25 key aroma-active compounds were further verified by aroma recombination, and their crucial role was confirmed in the formation of NG and HG aromas. Both NG and HG were dominated by lipid-derived aroma compounds, including aldehydes, ketones, and alcohols. Differences between the aromas of NG and HG were in the OAV levels. NG contained higher OAVs of aldehydes and displayed a more intense flavor of the green, fatty, citrus-like odor impressions. The higher OAVs of (*E*)-*β*-damascenone and 3-methyl-2,4-nonanedione were determined in HG due to the thermal reaction. Even though the drying processes of goji berries were different, the fatty acids, which indicated the nutritional values, were not significantly different. The results of this study indicated that hot-air drying could maintain a more characteristic goji flavor and less green and fatty flavor. (Furan) Fatty acids were determined for the first time for different dried goji berries. The differences in fatty acids between NG and HG were mainly due to the contributions of polyunsaturated fatty acids. HG composed fewer contributions of linoleic acid and *γ*-linolenic acid. Furan fatty acids are too weak to be detected in dried goji berries. The parameters and techniques of the commercial drying process of red goji berries were not open to the public, and some pre-treatments, including the osmotic dehydration [45], sodium carbonate, potassium hydroxide, ultrasonic, and sucrose ester pre-treatments [10,48], are usually used for the commercial drying of goji berries. The results of this work could provide a theoretical basis and reference value for post-harvest processing to promote the commercial value of goji products.

## Figures and Tables

**Table 1:** RIs on DB-5 and DB-FFAP capillary columns, odor quality, and the FD factors of aroma-active compounds in two dried red goji berries.

No. ^a^	Compound	RI ^b^		Odor Quality	FD Factor ^c^	
		DB-5	DB-FFAP		NG	HG
**1**	ethyl butanoate	801	1032	fruity	16	
**2**	hexanal	801	1078	green, grassy	256	256
**3**	methyl hexanoate	923	1181	fruity, musty	4	4
**4**	3-methyl-1-butanol	<800	1198	malty	16	2
**5**	(*Z*)-4-heptenal	900	1237	fish-like, train oil-like	2	1
**6**	octanal	1004	1283	citrus-like, green	16	4
**7**	1-octen-3-one	978	1295	mushroom-like	8	8
**8**	2-acetyl-1-pyrrolin ^d^	909	1314	popcorn-like, roasty	64	128
**9**	(*E*)-2-heptenal	958	1319	green apple-like, bitter almond-like	1	1
**10**	dimethyl trisulfide	973	1375	sulfuric, cabbage-like	2	1
**11**	nonanal	1106	1386	citrus-like, soapy	1	2
**12**	(*E*)-2-octenal	1060	1425	fatty, nutty	1	1
**13**	2-ethyl-3,5-dimethylpyrazine	1077	1436	earthy	8	8
**14**	1-octen-3-ol	981	1439	mushroom-like	1	1
**15**	ethyl cyclohexanoate ^d^	1136	1414	fruity, sweet	8	1
**16**	acetic acid	<700	1444	vinegar-like	512	128
**17**	(*E*,*E*)-2,4-heptadienal	1013	1490	fatty, floral	4	1
**18**	decanal	1208	1491	soapy, citrus-like	16	512
**19**	pentyl hexanoate	1278	1506	fruity, ethereal		8
**20**	3-isobutyl-2-methoxypyrazine ^d^	1177	1516	earthy, green bell pepper-like	4	2
**21**	(*E*)-2-nonenal	1161	1529	fatty, green	128	32
**22**	1-octanol	1070	1550	soapy, citrus-like, green	8	1
**23**	(*E*,*Z*)-2,6-nonadienal	1155	1581	cucumber-like	8	4
**24**	2-undecanone	1293	1590	soapy, green	32	
**25**	butanoic acid	<800	1621	sweaty	32	16
**26**	2-acetylpyrazine ^d^	1024	1625	popcorn-like, roasty	8	8
**27**	(*E*)-2-decenal	1264	1638	fatty, green	2	
**28**	phenylacetaldehyde	1047	1642	floral, honey-like	8	8
**29**	2-, and 3-methylbutanoic acid	857	1662	sweaty	128	64
**30**	*α*-terpineol	1199	1690	floral, citrus-like	1	1
**31**	(*E*,*E*)-2,4-nonadienal	1218	1697	fatty, green	4	4
**32**	3-methyl-2,4-nonanedione	1262	1715	hay-like, aniseed-like, fish-like	32	64
**33**	(*E*)-2-undecenal	1366	1747	soapy, metallic	2	2
**34**	(*E*,*E*)-2,4-decadienal	1322	1807	fatty, deep-fried	1	
**35**	(*E*)-*β*-damascenone	1382	1817	cooked apple-like	4	4
**36**	2-methoxyphenol	1087	1858	smoky, gammon-like	2	1
**37**	heptanoic acid	1075	1945	rancid, sweaty	1	32
**38**	(*E*)-3-hexenoic acid ^d^	986	1947	floral, cheesy	1	2
**39**	*cis*-4,5-epoxy-(*E*)-2-decenal	1369	1986	metallic	128	256
**40**	*trans*-4,5-epoxy-(*E*)-2-decenal	1384	1993	metallic	256	1024
**41**	phenol	975	2005	ink-like, phenolic	1	
**42**	4-hydroxy-2,5-dimethyl-3(2*H*)-furanone	1158	2033	caramel-like	1024	256
**43**	*p*-cresol	1072	2081	fecal, phenolic, horse stable-like	1	2
**44**	4-ethyl-2-methoxyphenol ^d^	1275	2032	smoky, gammon-like		8
**45**	nonanoic acid	1282	2158	moldy, pungent	8	16
**46**	eugenol	1357	2166	clove-like	2	2
**47**	4-ethylphenol	1164	2174	phenolic	128	128
**48**	2-methoxy-4-vinylphenol	1311	2197	smoky, clove-like	2048	2048
**49**	3-hydroxy-4,5-dimethyl-2(5*H*)-furanone	1100	2198	fenugreek-like, lovage-like	8	8
**50**	phenylacetic acid	1241	2561	honey-like, beeswax-like	64	64
**51**	vanillin	1401	2572	vanilla-like, sweet	2048	1024
**52**	3-phenylpropanoic acid	1346	2629	floral, cinnamon-like	4	4

^a^ The number was continuously given based on the orders of the retention indices on the DB-FFAP capillary column. ^b^ RI (retention index) was calculated according to the retention times and carbon numbers of the adjacent n-alkanes reference solution (C_6_-C_19_ for DB-5 capillary column and C_6_-C_26_ for DB-FFAP capillary column). ^c^ Flavor dilution (FD) factor was the highest dilution factor of the SAFE distillate when the aroma-active compound was perceivable on the sniffing port by using GC/O (DB-FFAP capillary column) during the AEDA analysis; NG, naturally dried red goji berries; HG, hot-air-dried red goji berries. ^d^ The compound was too trace that it was hard to get a clear MS spectrum, and the identification of the compound was carried out by comparing the RIs (on both DB-5 and DB-FFAP capillary columns), odor quality, and odor intensity with the authentic reference.

**Table 2:** Calibration curves, R^2^, and contents of the aroma-active compounds in two dried red goji berries.

No.	Compound	Quantifier Ion (*m/z*) ^a^	Calibration Curve ^b^	R^2^	NG ^c^		HG ^d^	
					Con.	RSD	Con.	RSD
**16**	acetic acid	60	y = 0.1199x − 0.0278	0.9996	320,000	14	170,000	11
**21**	(*E*)-2-nonenal	55	y = 0.9263x + 0.0776	0.9983	1400	13	250 **	13
**2**	hexanal	56	y = 0.2749x + 0.0211	0.9984	1200	14	1700 *	2.8
**6**	octanal ^e^	56	y = 0.8613x − 0.0218	1	1100	14	390 ***	12
**4**	3-methyl-1-butanol	55	y = 0.4732x − 0.076	0.9939	770	14	260 **	7.2
**45**	nonanoic acid	60	y = 0.1318x − 0.0313	0.9984	620	5.4	1800 **	7.6
**29**	2, and 3-methylbutanoic acid ^f^	60	y = 0.6866x − 0.1458	0.9998	590	10	390 **	6.0
**18**	decanal	57	y = 0.0528x − 0.0104	0.9975	220	3.7	1300 ***	6.1
**22**	1-octanol	56	y = 0.6225x − 0.0748	0.9993	210	6.6	4.8 **	3.2
**51**	vanillin ^g^	151	y = 0.9997x − 0.1974	0.9958	75	6.8	95 *	9.7
**11**	nonanal	57	y = 0.2182x − 0.0336	0.9999	39	7.7	18 **	2.7
**40**	*trans*-4,5-epoxy-(*E*)-2-decenal	68	y = 0.0884x − 0.0023	0.9978	38	3.0	53 *	9.0
**1**	ethyl butanoate	71	y = 0.2292x − 0.0161	0.9916	33	15	0.64 **	3.5
**32**	3-methyl-2,4-nonanedione	99	y = 0.1156x + 0.0086	0.9973	32	13	81 **	2.9
**39**	*cis*-4,5-epoxy-(*E*)-2-decenal	68	y = 0.0422x − 0.0013	0.9996	29	9.1	41 **	2.6
**25**	butanoic acid ^h^	73	y = 0.8392x + 0.1731	0.9952	25	3.4	22 **	1.6
**42**	4-hydroxy-2,5-dimethyl-3(2*H*)-furanone	128	y = 0.2055x + 0.0075	0.9925	24	4.9	6.5 **	2.1
**37**	heptanoic acid	60	y = 0.354x − 0.0846	0.9929	21	4.1	1100 **	9.7
**50**	phenylacetic acid ^i^	136	y = 0.964x + 1.6184	0.999	14	4.7	14	10
**5**	(*Z*)-4-heptenal	41	y = 0.1385x − 0.0331	0.9993	11	8.0	5.3 **	3.0
**14**	1-octen-3-ol	57	y = 0.9689x − 0.0926	0.9969	11	8.4	9.4	5.8
**24**	2-undecanone	58	y = 1.4768x − 0.1106	0.9925	10	12	1.7 **	14
**9**	(*E*)-2-heptenal	83	y = 0.33x − 0.0402	0.9908	9.1	14	6.4	12
**12**	(*E*)-2-octenal	55	y = 0.395x − 0.0353	0.9981	6.8	5.4	5.8 *	6.6
**27**	(*E*)-2-decenal	70	y = 0.316x − 0.0353	0.9928	6.0	6.1	0.96 **	14
**7**	1-octen-3-one	55	y = 0.9102x − 0.2433	0.9938	5.6	4.2	6.1	7
**36**	2-methoxyphenol	109	y = 0.7453x − 0.11	0.9924	5.5	11	1.2 ***	4.5
**48**	2-methoxy-4-vinylphenol	135	y = 0.7976x − 0.1984	0.9916	5.5	8.0	1.2 **	3.0
**17**	(*E*,*E*)-2,4-heptadienal	81	y = 1.0704x − 0.2174	0.9965	5.4	6.1	1.3 **	12
**47**	4-ethylphenol	107	y = 1.5506x − 0.0697	0.9996	4.6	13	1.9 **	10
**28**	phenylacetaldehyde	91	y = 1.8407x − 0.1792	0.9958	4.5	8.5	5.7	11
**3**	methyl hexanoate	74	y = 0.9783x − 0.2711	0.9912	4.4	12	5.4 *	2.6
**34**	(*E*,*E*)-2,4-decadienal	81	y = 1.5075x − 0.3252	0.9912	4.4	13	0.56 **	20
**38**	(*E*)-3-hexenoic acid	55	y = 0.1498x + 0.0073	0.9965	3.9	1.5	27 **	6.9
**41**	phenol	94	y = 1.3524x − 0.124	0.9982	2.8	12		
**30**	*α*-terpineol	59	y = 0.7084x − 0.0662	0.9981	1.7	0.83	0.09 ***	2.5
**31**	(*E*,*E*)-2,4-nonadienal	81	y = 1.8019x + 0.1319	0.9991	0.82	11	0.45 *	11
**35**	(*E*)-*β*-damascenone	69	y = 0.0259x − 0.0029	0.9983	0.75	15	1.8 *	12
**52**	3-phenylpropanoic acid	91	y = 0.1619x − 0.0517	0.9974	0.67	11	1.1 *	14
**13**	2-ethyl-3,5-dimethylpyrazine	135	y = 0.2879x + 0.0422	1	0.56	13	0.55	6.4
**49**	3-hydroxy-4,5-dimethyl-2(5*H*)-furanone	83	y = 0.0871x + 0.0034	0.9964	0.35	13	0.71 *	14
**23**	(*E*,*Z*)-2,6-nonadienal	70	y = 0.71x − 0.073	0.9943	0.31	2.0	0.21 **	14
**33**	(*E*)-2-undecenal	70	y = 0.439x − 0.094	0.9918	0.18	15	0.43 **	0.4
**46**	eugenol	164	y = 0.4707x − 0.0426	0.9977	0.06	16	0.06	8.2
**1**	dimethyl trisulfide	126	y = 0.9653x − 0.0892	0.9974	0.05	5.7	0.06	14
**43**	*p*-cresol	107	y = 1.2465x − 0.0692	0.9997	0.05	22	0.14 **	7.2
**19**	pentyl hexanoate	70	y = 0.6198x + 0.0138	0.9902			0.44	11

^a^ Quantifier ion was used for the quantitation in electron impact (EI) mode. ^b^ The curve was established by the peak area ratios and concentration ratios of the analytes and internal standards; the quantifier ion for the internal standard (1,2-dichlorobenzene) was selected as *m/z* 146. ^c^ NG, naturally dried red goji berries; Con., the average contents (µg/kg) of 2–4 samples; RSDs, relative standard derivations. ^d^ HG, hot-air-dried red goji berries; Con., the average contents (µg/kg) of 2–4 samples; RSDs, relative standard derivations; * indicates significantly different compared to NG with *p* < 0.05; ** indicates significantly different compared to NG with *p* < 0.01; *** indicates significantly different compared to NG with *p* < 0.001. ^e^ Octanal was quantified with d_16_-octanal as the internal standard (quantifier ion *m/z* 62). ^f^ A sum of 2, and 3-methylbutanoic acids was quantified with d_2_-3-methylbutanoic acid as the internal standard (quantifier ion *m/z* 63). ^g^ Vanillin was quantified with *d_3_*-vanillin as the internal standard (quantifier ion *m/z* 154). ^h^ Butanoic acid was quantified with *d_2_*-butanoic acid as the internal standard (quantifier ion *m/z* 74). ^i^ Phenylacetic acid was quantified with *d_2_*-phenylacetic acid as the internal standard (quantifier ion *m/z* 138).

**Table 3:** Odor thresholds (OTs) and odor activity values (OAVs) of aroma-active compounds in NG and HG.

No.	Compound	OT ^a^	OAV ^b^	
			NG	HG
**21**	(*E*)-2-nonenal	0.25	5800	1000
**5**	(*Z*)-4-heptenal	0.0087	1300	610
**32**	3-methyl-2,4-nonanedione	0.046	700	1800
**2**	hexanal	2.4	510	730
**7**	1-octen-3-one	0.016	350	380
**6**	octanal	3.4	340	110
**40**	*trans*-4,5-epoxy-(*E*)-2-decenal	0.22	170	240
**34**	(*E*,*E*)-2,4-decadienal	0.027	160	21
**39**	*cis*-4,5-epoxy-(*E*)-2-decenal	0.22	130	190
**35**	(*E*)-*β*-damascenone	0.006	130	290
**16**	acetic acid	5600	56	31
**1**	ethyl butanoate	0.75	45	<1
**45**	nonanoic acid	26 ^c^	24	71
**18**	decanal	9.3	23	140
**11**	nonanal	2.8	14	7
**31**	(*E*,*E*)-2,4-nonadienal	0.062	13	7
**27**	(*E*)-2-decenal	0.49 ^c^	12	2
**23**	(*E*,*Z*)-2,6-nonadienal	0.03	10	7
**36**	2-methoxyphenol	0.84	7	1
**10**	dimethyl trisulfide	0.0099	5	6
**4**	3-methyl-1-butanol	220	3	1
**13**	2-ethyl-3,5-dimethylpyrazine	0.28	2	2
**12**	(*E*)-2-octenal	4	2	2
**51**	vanillin	53	1	2
**29**	2-, and 3-methylbutanoic acid	490	1	<1
**38**	(*E*)-3-hexenoic acid	3.9	<1	7
**28**	phenylacetaldehyde	5.2	<1	1
**9**	(*E*)-2-heptenal	18 ^c^	<1	<1
**24**	2-undecanone	24 ^c^	<1	<1
**47**	4-ethylphenol	13	<1	<1
**42**	4-hydroxy-2,5-dimethyl-3(2*H*)-furanone	87	<1	<1
**48**	2-methoxy-4-vinylphenol	21 ^c^	<1	<1
**14**	1-octen-3-ol	45	<1	<1
**33**	(*E*)-2-undecenal	0.78	<1	<1
**49**	3-hydroxy-4,5-dimethyl-2(5*H*)-furanone	1.7	<1	<1
**50**	phenylacetic acid	68	<1	<1
**3**	methyl hexanoate	90	<1	<1
**46**	eugenol	1.8	<1	<1
**43**	*p*-cresol	3.9	<1	<1
**25**	butanoic acid	2400	<1	<1
**52**	3-phenylpropanoic acid	120	<1	<1
**30**	*α*-terpineol	1200	<1	<1
**41**	phenol	3400 ^c^	<1	<1
**44**	4-ethyl-2-methoxyphenol	50	<1	<1

^a^ OT (odor threshold) was obtained from the data in the water matrix in the Leibniz-LSB@TUM Odorant Database (https://www.leibniz-lsb.de/datenbanken/leibniz-lsbtum-odorant-database/odorantdb, accessed on 22 Apil 2024). ^b^ The OAV (odor activity value) was the ratio of the determined content and OT; NG, naturally dried red goji berries; HG, hot-air-dried red goji berries. ^c^ OT was obtained from the data in the water matrix in the in-house database.

**Table 4:** Contents and contributions of fatty acids in NG and HG.

Fatty Acid	Structure	Content (µg/kg)		µg Fatty Acid/mg of Fat		Contribution (%)	
		NG ^a^	HG ^b^	NG	HG	NG	HG
caprylic	8:0	0.47 ± 0.00	0.27 ± 0.01 ***	0.20 ± 0.02	0.12 ± 0.00 *	0.20 ± 0.02	0.10 ± 0.02 *
lauric	12:0	0.06 ± 0.01	0.11 ± 0.03	0.03 ± 0.01	0.05 ± 0.01	0.03 ± 0.00	0.04 ± 0.00 *
myristic	14:0	0.53 ± 0.11	0.75 ± 0.21	0.22 ± 0.06	0.33 ± 0.08	0.22 ± 0.02	0.27 ± 0.02
pentadecanoic	15:0	0.06 ± 0.01	0.09 ± 0.02	0.03 ± 0.01	0.04 ± 0.01	0.03 ± 0.00	0.03 ± 0.00 *
palmitic	16:0	25 ± 4.0	33 ± 8.1	11 ± 2.4	15 ± 3.0	11 ± 0.32	12 ± 0.37
heptadecanoic	17:0	0.52 ± 0.07	0.53 ± 0.13	0.22 ± 0.05	0.24 ± 0.05	0.22 ± 0.00	0.19 ± 0.01
isomer of stearic	isomer of 18:0	0.23 ± 0.03	0.32 ± 0.08	0.10 ± 0.02	0.15 ± 0.03	0.10 ± 0.00	0.12 ± 0.00 *
stearic	18:0	5.7 ± 0.75	7.2 ± 1.45	2.4 ± 0.49	3.2 ± 0.51	2.4 ± 0.01	2.6 ± 0.04 **
arachidic	20:0	1.5 ± 0.25	1.6 ± 0.40	0.65 ± 0.15	0.72 ± 0.15	0.65 ± 0.02	0.58 ± 0.02 *
henicosanoic	21:0	0.16 ± 0.03	0.16 ± 0.05	0.07 ± 0.02	0.07 ± 0.02	0.07 ± 0.01	0.06 ± 0.01
behenic	22:0	2.9 ± 0.52	3.0 ± 0.79	1.2 ± 0.31	1.3 ± 0.30	1.2 ± 0.06	1.1 ± 0.06
tricosanoic	23:0	0.17 ± 0.02	0.08 ± 0.00 *	0.07 ± 0.01	0.04 ± 0.00 *	0.07 ± 0.00	0.03 ± 0.01 **
lignoceric	24:0	2.24 ± 0.39	3.3 ± 0.71	0.95 ± 0.23	1.5 ± 0.26	0.95 ± 0.04	1.2 ± 0.00 **
ΣSFA		40 ± 6.3	52 ± 12	17 ± 3.9	23 ± 4.5	17 ± 0.47	19 ± 0.32
***cis***-10-pentadecenoic	15:1*n-5*	0.26 ± 0.06	0.44 ± 0.11	0.11 ± 0.03	0.20 ± 0.04	0.11 ± 0.01	0.16 ± 0.01 *
palmitoleic	16:1*n-7*	1.06 ± 0.20	1.6 ± 0.43	0.45 ± 0.12	0.72 ± 0.16	0.44 ± 0.03	0.59 ± 0.03 *
oleic	18:1*n-9c*	32 ± 4.0	37 ± 7.3	14 ± 2.7	16 ± 2.6	14 ± 0.07	13 ± 0.22
elaidic	18:1*n-9t*	2.1 ± 0.29	2.7 ± 0.57	0.87 ± 0.19	1.2 ± 0.20	0.87 ± 0.01	0.99 ± 0.01 **
***cis***-11-eicosenoic	20:1*n-9*	0.20 ± 0.05	0.28 ± 0.04 *	0.08 ± 0.03	0.13 ± 0.01 **	0.08 ± 0.01	0.10 ± 0.01 **
linoleic	18:2*n-6*	140 ± 16	150 ± 32	58 ± 11	69 ± 11	58 ± 0.69	56 ± 0.54 **
***γ***-linolenic	18:3*n-6*	9.2 ± 1.1	9.7 ± 2.6	3.9 ± 0.76	4.3 ± 0.96	3.9 ± 0.03	3.5 ± 0.17 *
***α***-linolenic	18:3*n-3*	15 ± 2.6	18 ± 4.6	6.4 ± 1.5	8.0 ± 1.7	6.3 ± 0.27	6.5 ± 0.25
ΣUFA		200 ± 24	220 ± 47	83 ± 16	100 ± 17	83 ± 0.46	81 ± 0.32
sum		240 ± 31	280 ± 60	100 ± 20	120 ± 21	100	100

^a^ The content of NG (naturally dried red goji berries) is the average content (µg/kg) of the duplicates with the correction of the internal standards (myristic acid ethyl ester and 10,11-dichloroundecanoic acid) and a Supelco 37 component fatty acid methyl ester (FAME) reference solutions. ^b^ The content of HG (hot-air-dried red goji berries) is the average content (µg/kg) of the duplicates with the correction of the internal standards (myristic acid ethyl ester and 10,11-dichloroundecanoic acid) and a Supelco 37 component fatty acid methyl ester (FAME) reference solutions; the significant differences between NG and HG were analyzed by a *t*-test; * indicates *p* < 0.05; ** indicates *p* < 0.01; *** indicates *p* < 0.001.

**Table 5:** Structures, proposed precursors, and mechanisms of represented aldehydes in both dried red goji berries, according to ref. [27,28,36,42].

No.	Aldehyde	Content Ratio of NG and HG	Potential Precursor ^a^	Content Ratio of NG and HG	Hydroperoxide	Proposed Mechanism ^b^
**21**	(*E*)-2-nonenal	5.7	LA (18:2*n-6*)	0.89	LA-10-OOH	AO, PO, EO
			GLA (18:3*n-6*)	0.95	GLA-9-OOH	
**5**	(*Z*)-4-heptenal	2.2	ALA (18:3*n-3*)	0.83	ALA-10-OOH	PO, EO
**2**	hexanal	0.70	LA (18:2*n-6*)	0.89	LA-12-OOH	AO, PO, EO
			GLA (18:3*n-6*)	0.95	GLA-11-OOH	
**34**	(*E*,*E*)-2,4-decadienal	7.9	LA (18:2*n-6*)	0.89	LA-9-OOH	AO, PO, EO
			GLA (18:3*n-6*)	0.95	GLA-8-OOH	
**6**	octanal	2.8	OA (18:1*n-9*)	0.75	OA-11-OOH	AO
**40**, **39**	*trans*- and *cis*- 4,5-epoxy-(*E*)-2-decenal	0.72	LA (18:2*n-6*)	0.89	LA-13-OOH	AO, PO, EO
**18**	decanal	0.17	OA (18:1*n-9*)	0.75	OA-8-OOH	AO
**11**	nonanal	2.1	OA (18:1*n-9*)	0.75	OA-10-OOH	AO, PO
**31**	(*E*,*E*)-2,4-nonadienal	1.8	ALA (18:3*n-3*)	0.83	ALA-10-OOH	PO, EO
**27**	(*E*)-2-decenal	6.2	OA (18:1*n-9*)	0.75	OA-9-OOH	AO, PO
**12**	(*E*)-2-octenal	1.2	PA (16:1*n-7*)	0.65	PA-9-OOH	AO, PO

^a^ The precursor was the proposed precursor. ^b^ AO, autooxidation; PO, photooxidation; EO, enzyme-catalyzed oxidation.

## Data Availability

The original contributions presented in the study are included in the article; further inquiries can be directed to the corresponding authors.

## Supplementary Materials

The following supporting information can be downloaded at: https://www.mdpi.com/article/10.3390/foods14132388/s1, Figure S1: The mass spectrum of (**a**) 11-(3,4-dimethyl-5-pentylfuran-2-yl)-undecanoic acid methyl ester (11D5-ME) and (**b**) 9-(5-pentylfuran-2-yl)-nonanoic acid methyl ester (9F5-ME) in dried red goji berries.
