# Supplementary material for: Fatty Acids Are Responsible for the Discrepancy of Key Aroma Compounds in Naturally Dried Red Goji Berries and Hot-Air-Dried Red Goji Berries
Source: Foods. 2025 Jul 6;14(13):2388. doi: 10.3390/foods14132388 (PMC12248973; doi:10.3390/foods14132388)
Supplement: Supplementary file 1 [file foods-14-02388-s001.zip › foods-3654252-supplementary.pdf]

1. Sample preparation and transesterification of furan fatty acids (FuFAs)

For analyzing FuFAs, the protocol was according to the previous description (Müller et al., 2020) with slight adjustments. The freeze-dried and grounded goji powder (~100 mg) in a brown glass tube was supplemented with 3 mL fresh prepared 1% sulphuric acid methanol solution and set into an argon atmosphere to protect FuFAs. After being treated in an ultrasonic bath for 3 min, the test tube was transesterified at 80 °C in a sand bath for 2 h and treated in an ultrasonic bath for 1 min every 30 min. After transesterification, the sample was cooled on ice and then added *n*-hexane (2 mL), distilled water (1 mL), and saturated sodium chloride solution (1 mL). After shaking for 15 sec, the organic phase was transferred into a 1.5 mL brown glass vial and stored at -20 °C. Prior to measurement, the sample solution was 25 times diluted and supplemented with internal standard (14:0-EE, 0.01 mg/mL). All samples were prepared in duplicate.

2. Instrumental analysis of FuFAs

Transesterified FuFAs were measured according to Müller et al. (2020) with slight modifications. A trace 1310 Thermo GC (Waltham, MA, USA) equipped with an HP-5MS UI capillary column (5% phenyl, 95% methyl polysiloxane, 30 m × 0.25 mm i.d., 0.25 µm film thickness, Agilent, Waldbronn, Germany) was linked to a triple quadrupole MS (TSQ 8000; Thermo Scientific, Waltham, MA, USA). An aliquot of the sample (1 µL) was injected in splitless mode by a Thermo Triplus RSH autosampler (Thermo Scientific, Waltham, MA, USA). The inlet temperature was held at 50 °C for 0.2 min and elevated for three steps: (i) elevated to 100 °C at 14.5 °C/sec and held for 1 min, (ii) elevated to 250 °C at 14.5 °C/sec and held for 2 min, and (iii) elevated to 300 °C at 14.5 °C/sec and held for 2 min. The oven temperature was held at 60 °C for 1 min, heated to 180 °C at 13 °C/min, heated to 250 °C at 3 °C/min, heated to 300 °C

at 20 °C/min, and held at 300 °C for 5 min. The helium flow rate was 1.0 mL/min. The temperatures of the transferline and ion source were kept at 270 °C. The MS was recorded in both full scan mode (m/z 60-550) and SIM mode, as mentioned in Vetter et al. (2016).

3. Determination of the concentration of labeled references

The concentrations of d_2-butanoic acid, d_2-3-methylbutanoic acid, d_{16}-octanal, d_2-phenylacetic acid, and d_3-vanillin were analyzed by a Clarus 400 gas chromatograph (PerkinElmer, UK) in connection with an autosampler (Perkin Eimer, UK). The sample was injected and flushed onto the DB-5 fused silica capillary column (30 m × 0.25 mm inner diameter, 0.25 µm film thickness). The oven temperature was held at 40 °C for 2 min, ramped to 230 °C at 6 °C/min, and held for 5 min. At the end of the column, the effluent was analyzed by flame ionization detector (FID), which was held at 250 ˚C. The detector gas was 45 mL/min hydrogen and 450 mL/min air. A response factor was determined by analyzing mixtures of the amount of their respective unlabeled references and methyl octanoate in the ratio of 1:1. The response factor of unlabeled reference and methyl octanoate was used for the calculation of the concentration of labeled compound.

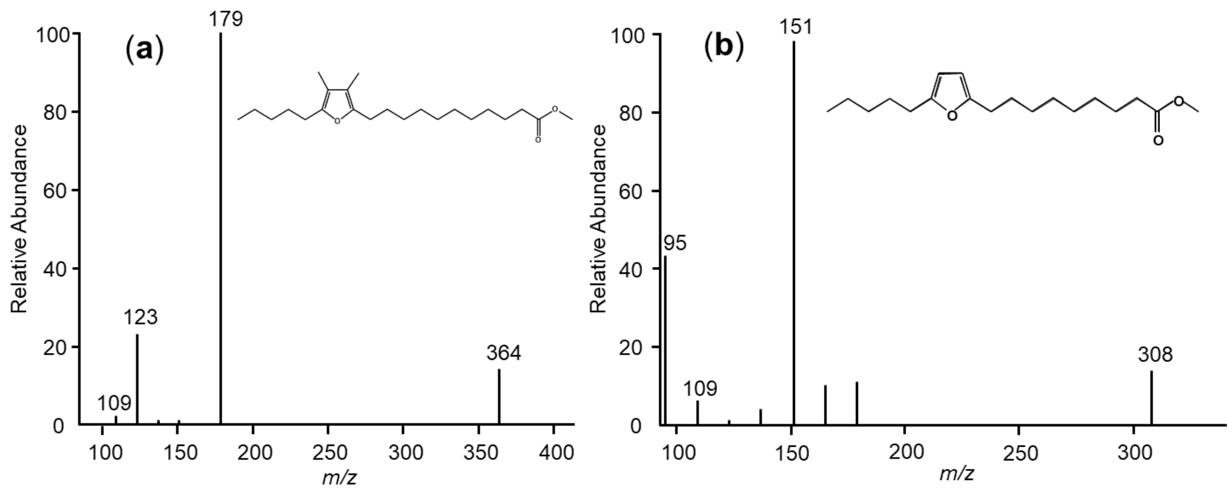

Fig. S1. The mass spectrum of (**a**) 11-(3,4-dimethyl-5-pentylfuran-2-yl)-undecanoic acid methyl ester (11D5-ME) and (**b**) 9-(5-pentylfuran-2-yl)-nonanoic acid methyl ester (9F5-ME) in dried red goji berries
